# Diversity of Rickettsia species in collected ticks from Southeast Iran

**DOI:** 10.1186/s12917-024-04142-4

**Published:** 2024-06-27

**Authors:** Ali Qorbani, Mohammad Khalili, Saeidreza Nourollahifard, Ehsan Mostafavi, Mehrdad Farrokhnia, Saber Esmaeili

**Affiliations:** 1 Department of Pathobiology, Faculty of Veterinary Medicine, Shahid Bahonar University of Kerman, Kerman, Iran; 2 National Reference Laboratory for Plague, Tularemia and Q fever, Research Centre for Emerging and Reemerging infectious diseases, Pasteur Institute of Iran, Akanlu, Hamadan, Kabudar Ahang Iran; 3 Department of Epidemiology and Biostatistics, Research Centre for Emerging and Reemerging infectious diseases, Pasteur Institute of Iran, Tehran, Iran; 4 Department of internal Medicine, School of Medicine, Kerman University of Medical Sciences, Kerman, Iran

**Keywords:** Rickettsiosis, *Rickettsia*, Ticks, Iran, Molecular Epidemiology

## Abstract

*Rickettsia* occurs worldwide and rickettsiosis is recognized as an emerging infection in several parts of the world. Ticks are reservoir hosts for pathogenic *Rickettsia* species in humans and domestic animals. Most pathogenic *Rickettsia* species belong to the spotted Fever Group (SFG). This study aimed to identify and diagnose tick fauna and investigate the prevalence of *Rickettsia* spp. in ticks collected from domestic animals and dogs in the rural regions of Kerman Province, Southeast Iran. In this study, tick species (fauna) were identified and 2100 ticks (350 pooled samples) from two genera and species including *Rhipicephalus linnaei* (1128) and *Hyalomma deteritum* (972) were tested to detect *Rickettsia* genus using Real-time PCR. The presence of the *Rickettsia* genus was observed in 24.9% (95%CI 20.28–29.52) of the pooled samples. Sequencing and phylogenetic analyses revealed the presence of *Rickettsia aeschlimannii* (48.98%), *Rickettsia conorii israelensis* (28.57%), *Rickettsia sibirica* (20.41%), and *Rickettsia helvetica* (2.04%) in the positive samples. The results showed a significant association between county variables and the following variables: tick spp. (*p* < 0.001), *Rickettsia* genus infection in ticks (*p* < 0.001) and *Rickettsia* spp. (*p* < 0.001). In addition, there was a significant association between tick species and host animals (dogs and domestic animals) (*p* < 0.001), *Rickettsia* spp infection in ticks (*p* < 0.001), and *Rickettsia* spp. (*p* < 0.001). This study indicates a high prevalence of *Rickettsia* spp. (SFG) in ticks of domestic animals and dogs in rural areas of Kerman Province. The health system should be informed of the possibility of rickettsiosis and the circulating species of *Rickettsia* in these areas.

## Introduction

Although vector-borne diseases (VBDs) are globally prevalence, they are mostly reported in tropical and subtropical countries. The prevalence of these diseases depends on human and natural factors, such as climatic conditions and the movement of humans and animals. This makes their control and treatment difficult, especially in poor countries and areas where access to the health care system is limited.

Rickettsiosis or diseases caused by *Rickettsia* species represents a very important group because of the emergent character of the illness [1]. The *Rickettsiaceae* family includes small Gram-negative obligate intracellular pleomorphic bacteria. *Rickettsia* can be transmitted to animals and humans by hematophagous arthropods, causing specific zoonotic diseases, termed rickettsioses. The main vectors are ticks, although the pathogen can also be transmitted by other arthropods such as fleas, lice, or mites [1]. *Rickettsia* bacteria were divided into four groups based on the new *Rickettsia* genus classification: the spotted fever group (including *R. conorii, R. rickettsia*, and several others), typhus group (i.e. *R. typhi* and *R. prowazekii*), and ancestral group (including *R. Canadensis, R. bellii* nonpathogenic are known), and transitional group (including *R. felis*, *R. australis*, and *R. akari*). Many novel *Rickettsia* clades have been discovered in a variety of new hosts, including amoebae, insects, and leeches, providing a broader view of the evolution of *Rickettsia* [2].

In recent years, rickettsial infection in humans, animals, and ticks have been reported in most of the various countries in the Middle East Countries [3, 4]. Limited information is available on *Rickettsia* spp in Iran. In a study to identify *Rickettsia* species in ticks collected from sheep in the Khuzestan province, Southwest Iran, the tick species were identified as *Hyalomma marginatum*, *Hyalomma anatolicum*, *Hyalomma dromedarii*, *Hyalomma schulzei*, *Rhipicephalus bursa*, and *Rhipicephalus turanicus*. *Rickettsia* spp. were observed in 50% of ticks collected (50%). Sequencing and phylogenetic analyses revealed the presence of *Rickettsia aeschlimannii* (60%), *Rickettsia massiliae* (30%), and *Rickettsia conorii* (10%) in infected ticks [5].

In 2017–2018, five cases of human Mediterranean spotted fever (MSF) infection (caused by *R. conorii*) were reported in southeast Iran [6]. Limited information is available on the prevalence of *Rickettsia* in humans, domestic animals, and vectors. Further investigation is required to understand the epidemiology of this disease in Iran. Screening ticks for disease-causing pathogens provides useful epidemiological information on their distribution and the prevalence of pathogens that pose veterinary and medical health risks. The present study aimed to investigate the possible circulation of *Rickettsia* species and identify the variables associated with ticks infesting ticks collected from rural areas of southeastern Iran.

## Materials and methods

### Ethical code

The ethical code (IR. UK. VETMED. REC. 1399, 025) was obtained from the Ethics Committee of Shahid Bahonar University of Kerman. In addition, for the collection of ticks, verbal permission was obtained from the domestic animal owners.

### Study area

**Fig. 1:**
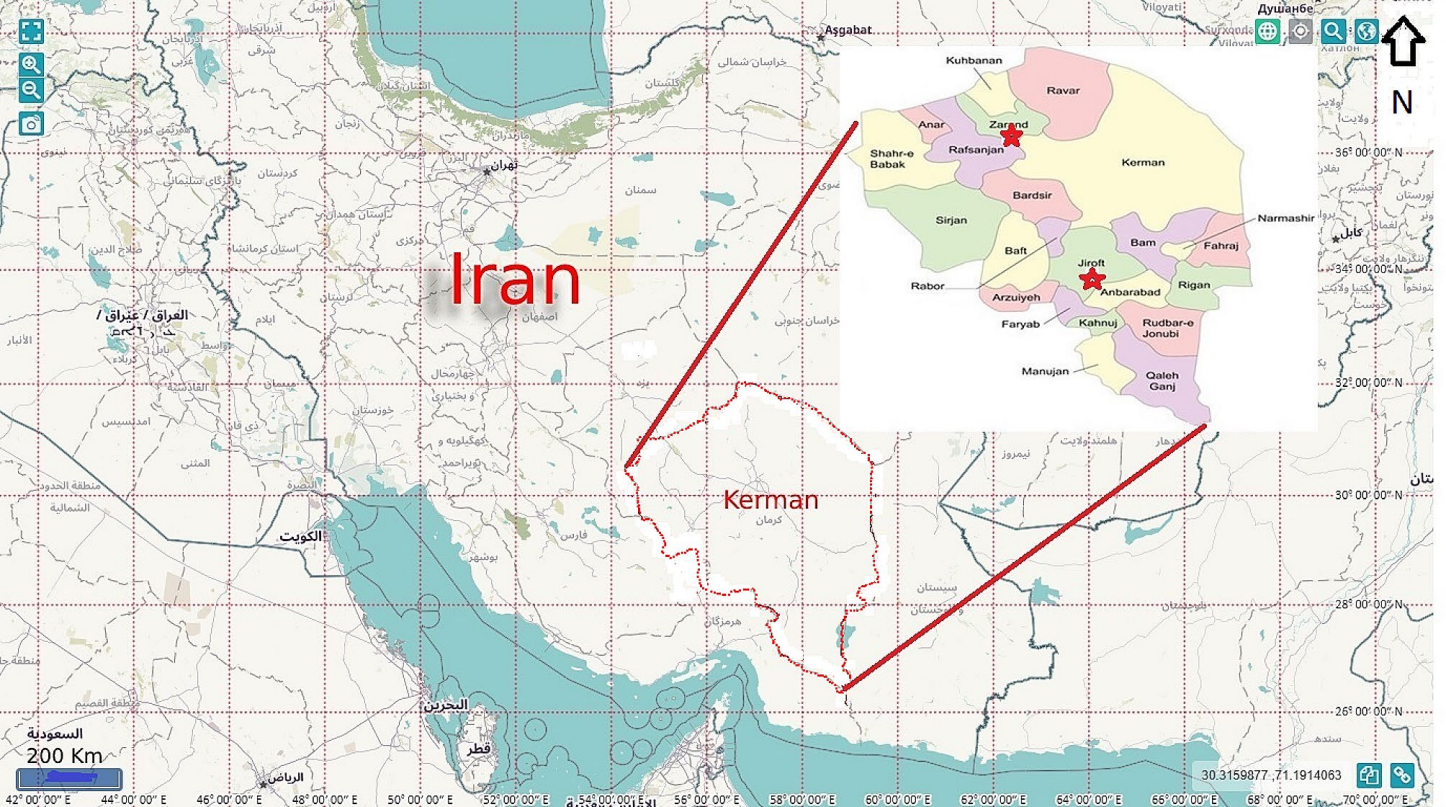
Geographical map of Jiroft and Zarand counties, located in Kerman Province, southeastern Iran. The counties studied are indicated with asterisks (*). The map was designed based on the authors

This study was carried out in Kerman Province in southeastern Iran in 2021 (January-September). The tick samples used in this study were collected from sheep, goats, cattle, and dogs from two counties in Kerman Province (Jiroft and Zarand). Kerman Province has a tropical climate, with an area of 182.301 km2 and a population of over 3 million people. Ticks of domestic animals and dogs were performed on farms in the villages of Zarand County (located in the northwestern part of Kerman Province with a population of 138,000, semi-arid climate, average annual precipitation of 140 mm, a height of 1664 m above sea level, and geographical location of 30.8 ^0^N and 56.58 ^0^E) and villages of Jiroft County (located in the southern part of Kerman Province with a population of 309,000, warm weather, average annual precipitation of 220 mm, height of 860 m above sea level, and geographical location of 28.91 ^0^N and 57.66 ^0^E) (Fig. 1).

### Tick collection and identification

In this study, ticks were collected between January and September 2021. First, ten villages were randomly selected from each county. Twenty farms in each village were randomly selected and included in this study. A total of 400 farms were included in this study. Tick specimens were collected from sheep, goats, cattle, and dogs at the sampling sites (farms) and verbal consent was obtained from animal handlers before examining their domestic animals for ticks. Using blunt forceps, ticks were collected (from the abdomen, neck, internal sides of the rear legs, tail, and ear) and placed into labeled vials containing 70% ethanol. The ticks were then transported to the laboratory for identification under a light stereomicroscope (Olympus, Japan). All ticks were morphologically identified using taxonomic keys [7, 8]. The specimens were pooled according to the species, sex, study site, and host animal. The pooled samples consisted of six adult ticks (three males and three females), and were grouped into 350 pools: 180 pools from Zarand County and 170 pools from Jiroft. The tick specimens were then stored at -20 °C for further examination.

### Extraction of tick nucleic acids

The DNA was extracted using the potassium acetate method [9]. Briefly, pooled ticks were homogenized in liquid nitrogen and sterile PBS, washed again in 70% ethanol, rinsed with sterile water, and dried. The ticks were frozen with liquid nitrogen and disrupted mechanically using 1.5 mL plastic microtubes with a pestle. Initially, 500 µL of lysis buffer [0.1 M Tris-HCl (pH 8.25), 0.05 M EDTA, 0.2 M sucrose, 0.5% SDS] and 20 µL proteinase K (10 mg/mL) were added to each tick lysate. The suspensions were incubated overnight at 56 °C. Next, 120 µL of 5 M potassium acetate was added to each sample and incubated on ice for 10 min. The samples were centrifuged at 12,000 xg for 10 min, and the supernatants were collected. For nucleic acid precipitation, 35 µL of 4 M sodium acetate, 0.25% acrylamide mix, and 1.0 mL of absolute ethanol were added to each supernatant, which was then incubated for 10 min at -20 °C, followed by centrifugation at 12,000 xg for 20 min. The 1.5 mL plastic microtubes were washed with 500 µL 70% ethanol and air-dried at room temperature. Finally, the extracts were resuspended in 75 µL of 1X TE buffer (1 mM Tris-HCl pH 8.0, 1 mM EDTA) and stored at -20 °C until use.

### Detection of the *Rickettsia* genus

DNA extracted from ticks was analyzed to detect the *gltA* gene of the *Rickettsia* genus using Real-Time PCR. The 20 µLreactions contained, 10 µL commercial master mix (RealQ Plus 2x Master Mix Ampliqon, Denmark), 2.5 µL template DNA, 900 nmol (0.3 µL) of forward and reverse primers (Table 1) [10], and sterile distilled water to final volume (6.9 µL). *R*. *conorii* DNA (Amplirun, Vircell) and distilled water were included in all assays as positive and negative controls (2.5 µL), respectively. Amplification was performed in a Light Cycler 96 system (Germany) programmed for 10-min activation at 95 ˚C, followed by 45 cycles at 95 ˚C for 15 s, and 60 ˚C for 60 s. Quantitative analysis was performed using Rotor-Gene Q Series software, and readings were taken at the end of each cycle in green color at 60 ˚C. Samples with a cycle threshold (Ct) value lower than 37 and a suitable melting curve (73 ± 0.5 °C) were considered positive for *Rickettsia* spp. [10].

**Table 1:** The primers used for the detection of the *Rickettsia* genus (*gltA*)

Primer name	5′-primer sequences-3′	Target locus	Amplicon, bp	Reference
PanRick-Forward	ATAGGACAACCGTTTATTT	Citrate synthase (*gltA*)	70	[11]
PanRick-Reverse	CAAACATCATATGCAGAAA			

### Determination of *Rickettsia* species

To select suitable samples and for final confirmation, the positive samples were sent to the Epidemiology Laboratory of the Pasteur Institute of Iran. Samples were tested using Taqman Real-time PCR assay (16 S rRNA) for confirmation of *Rickettsia* infection (Table 2) [12]. Samples with a cycle threshold (Ct) ≤ 30 in Taqman Real-time PCR assay were selected for the identification of *Rickettsia* species.

**Table 2:** The primers and probe used for the detection of the *16 S rRNA Rickettsia* gene using Taqman Real-time PCR assay

Primer name	5′-primer sequences-3′	Target locus	Amplicon, bp	Reference
Forward	5’-CGCAACCCTYATTCTTATTG-3’	*16 S rRNA*	149	[12]
Reverse	5’-CCTCTGTAAACACCATTGTAGCA-3’			
probe	6-FAM-TAAGAAAACTGCCGGTGATAAGCCGGAG-TAMRA			

Using conventional PCR, *Rickettsia* species were determined by *g1tA* and *ompA* gene amplification. The primers used for *g1tA* and *ompA* gene amplification are shown in Table 3 [13].

**Table 3:** The primers used for *g1tA* and *ompA* gene amplification

Primer name	5′-primer sequences-3′	Target locus	Amplicon, bp	Reference
*g1tA*-Forward	5’-GCTCTTCTCATCCTATGGCTATTAT-3’	*gltA*	834 bp	[13]
*g1tA*-Reverse	5’-CAGGGTCTTCRTGCATTTCTT-3’			
*ompA*-Forward	5’-ATGGCGAATATTTCTCCAAAA-3’	*ompA*	632 bp	[13]
*ompA*-Reverse	5’-GTTCCGTTAATGGCAGCATCT-3’			

The PCR products for each gene were sequenced (Genomin Co, Tehran, Iran). The sequences were analyzed using Chromas version 2.6.6. Finally, the *g1tA* and *ompA* gene sequences based on different *Rickettsia* spp. in the GenBank database were extracted, and phylogenetic analysis was performed using MEGA X (version 10.1).

### Statistical analysis

Data analysis was performed using SPSS software (version 26). The prevalence of qualitative data was estimated using descriptive statistics (95% CIs). Moreover, to evaluate the effect and statistical correlation of the variables, the Chi-square test was used for data analysis. Statistical significance was set at *P* < 0.05.

## Results

In this study, 2100 adult ticks (350 pools) were examined using molecular methods. After morphological examination, ticks were pooled according to species, sex, sampling location, and animal species in which they were collected. There were 1050 male ticks (50%) and 1050 female ticks (50%). A total of 1890 (315 pools = 90%) were collected from domestic livestock (cattle, sheep, and goats) and 210 (35 pools = 10%) were collected from dogs. In the present study, we identified two tick species using morphological keys. They were classified into two genera, *Hyalomma deteritum*, and *Rhipicephalus linnaei*, with the highest percentages of *Rhipicephalus linnaei* (1128 ticks = 53.71%) and *Hyalomma deteritum* (972 ticks = 46.29%) (Table 4).

**Table 4:** The population and characteristics of ticks collected from Zarand and Jiroft counties

County	Host animals	species Tick	No, of ticks in each pool	Ticks sex, in each pool	No, of pools (%)	Total (%)
**Jiroft**	cattle, sheep, and goats	*Rhipicephalus linnaei*	6	3 males and 3 females	153 (90%)	170 (48.6%)
**Jiroft**	dogs	*Rhipicephalus linnaei*	6	3 males and 3 females	17 (10%)	
**Zarand**	cattle, sheep, and goats	*Hyalomma deteritum*	6	3 males and 3 females	162 (90%)	180 (51.4%)
**Zarand**	dogs	*Rhipicephalus linnaei*	6	3 males and 3 females	18 (10%)	

### *Rickettsia* detection by real-time PCR

In Kerman Province, of the 350 DNA pooled samples tested by Real-Time PCR, 87 pools (24.9%; 95%CI 20.28–29.52) were positive for *Rickettsia*. Among 180 DNA pooled samples from Zarand County, 70 pools (38.90%; 95%CI 33.70–44.10) were positive for *Rickettsia*, and in Jiroft County among 170 DNA pooled samples, 17 pools (10%; 95%CI 6.80–13.20) were positive for *Rickettsia* (Table 5). *Hyalomma deteritum* had a greater percentage of positive pools (37.66%), and *Rhipicephalus linnaei* had a lower percentage of positive pools (13.80%) (Table 6). According to the number of positive pools in each county (38.90% in Zarand County and 10% in Jiroft County), rickettsial infection was significantly higher in ticks from Zarand County than in those from Jiroft County (*P <* 0.001). There was no statistically significant difference in *Rickettsia* infection between the host animals variable (animal species) and the positive results of tick infection with the *Rickettsia* variable (*P* = 0.076).

**Table 5:** Prevalence of *Rickettsia* in ticks by counties

County	Jiroft				Zarand				
Animal	**dogs**	**cattle**	**sheep**	**goats**	**dogs**	**cattle**		**sheep**	**goats**
No of animal	100	100	450	300	100	100	500		300
No of ticks N (%)	102 (10%)	118 (11.57%)	475 (46.56%)	325 (31.87%)	108 (10%)	105 (9.70%)	530 (49.10%)		337 (31.20%)
Tick species	*Rh. linnaei*	*Rh. linnaei*	*Rh. linnaei*	*Rh. linnaei*	*Rh. linnaei*	*H. deteritum*	*H. deteritum*		*H. deteritum*
Prevalence of *Rickettsia* in ticks N (%)	102 (10%)				420 (38.90%)				

**Table 6:** The population of ticks collected in the studied counties, and the prevalence of positive tick pools for the *Rickettsia* genus in 2021

Genus	Species	No. of collected ticks in each county Total *N* (%)		Number of tested pools	No. of positive pools for Rickettsia spp. (%)
		Jiroft *N* (%)	Zarand *N* (%)		
*Rhipicephalus*	*Rh. linnaei*	170 (100%)	18 (10%)	188	26 (13.80%)
*Hyalomma*	*H. deteritum*	0	162 (90%)	162	61 (37.66%)

### Identification and phylogenetic analysis of *Rickettsia* species

A total of 49 pool samples positive for *Rickettsia* were selected for species identification in such a way that the selected samples included different tick species from all studied counties and hosts. In addition, the load of *Rickettsia* DNA (CT ≥ 30) was considered in sample selection for the phylogeny survey. Based on the results of sequence BLAST in GenBank and phylogenetic analysis, four distinct species were identified from 49 sequenced *Rickettsia gltA* and *ompA* samples, the majority of which were *R. aeschlimannii* (*n* = 24, 48.98%) and *R. conorii israelensis* (*n* = 14, 28.57%). Other *Rickettsia* species identified in the present study included *R. sibirica* (*n* = 10, 20.41%) *and R. helvetica* (*n* = 1, 2.04%) (Table 7).

**Table 7:** The *Rickettsia* species in association with tick species and host animal

Rickettsia species	Host animal	Tick species	Total number of ticks (pools)	Positive samples (pools)	Prevalence of Rickettsia species in the positive samples (49)
*R. conorii israelensis*	cattle, sheep, and goats	*Rh. linnaei*	153	10	14 (28.57%; 95%CI 15.92–41.22)
	dogs	*Rh. linnaei*	35	4	
*R. sibirica*	cattle, sheep, and goats	*H. deteritum*	162	9	10 (20.41%; 95%CI 9.12–31.70)
	dogs	*Rh. linnaei*	35	1	
*R. aeschlimannii*	cattle, sheep, and goats	*H. deteritum*	162	20	24 (48.98%; 95%CI 34.99–62.97)
	dogs	*Rh. linnaei*	35	4	
*R. helvetica*	cattle, sheep, and goats	*H. deteritum*	162	1	1 (2.04%; 95%CI 0–6)
	dogs	*Rh. linnaei*	35	0	

In this study, *R. conorii israelensis* infection was detected in the ticks (*Rh. linnaei*) from different hosts (cattle, sheep, goats, and dogs) in Jiroft County. *R. aeschlimannii* and *R. sibirica* were identified in ticks (*H. deteritum*) collected from different hosts (cattle, sheep, goats, and dogs) in Zarand County. *R. helvetica* infection was detected in *H. deteritum* ticks isolated from cattle, sheep, and goats in Zarand County (Table 7).

**Fig. 2:**
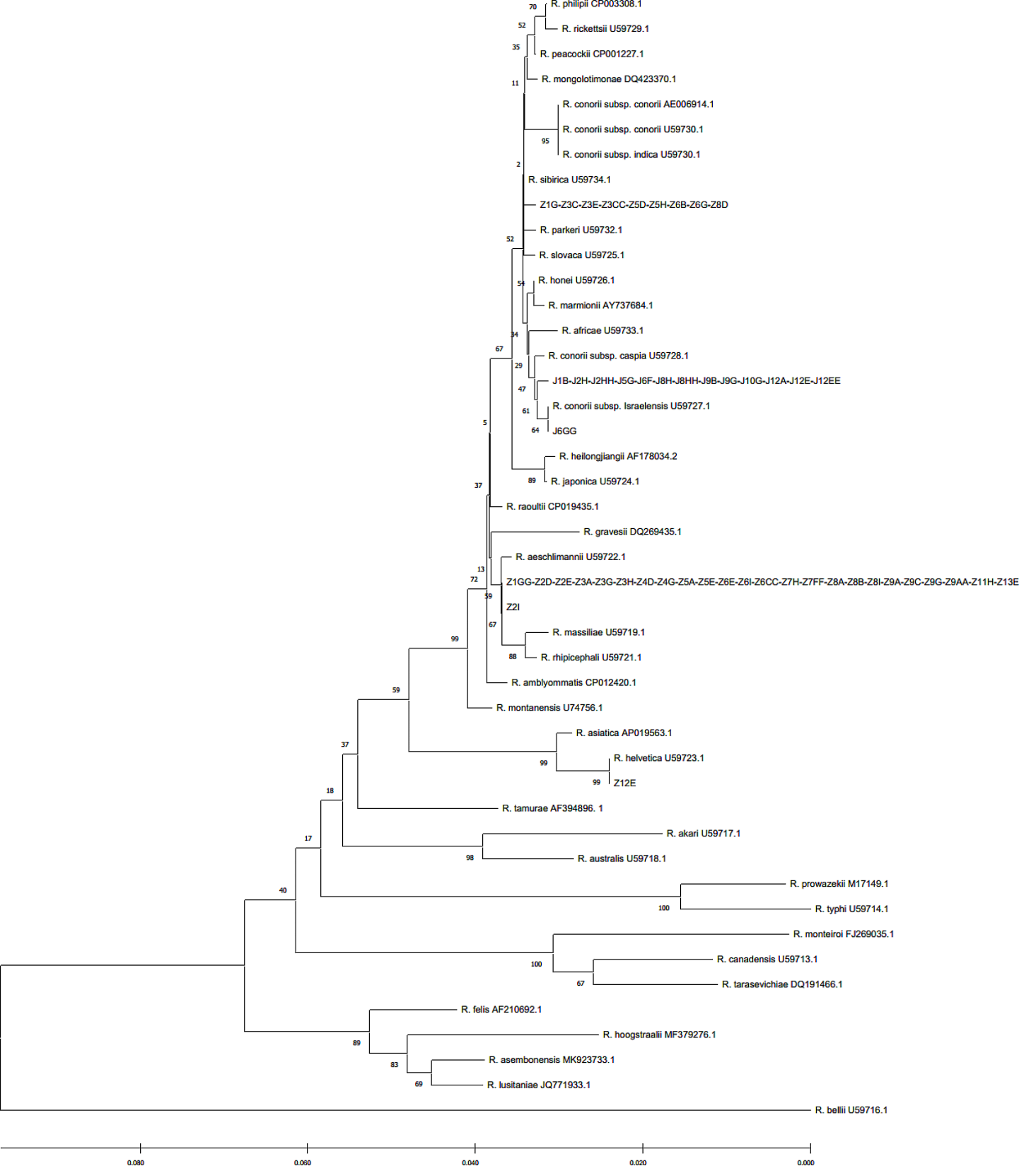
Phylogenetic tree diagram of *gltA* gene, extracted from bioinformatics analysis

**Fig. 3:**
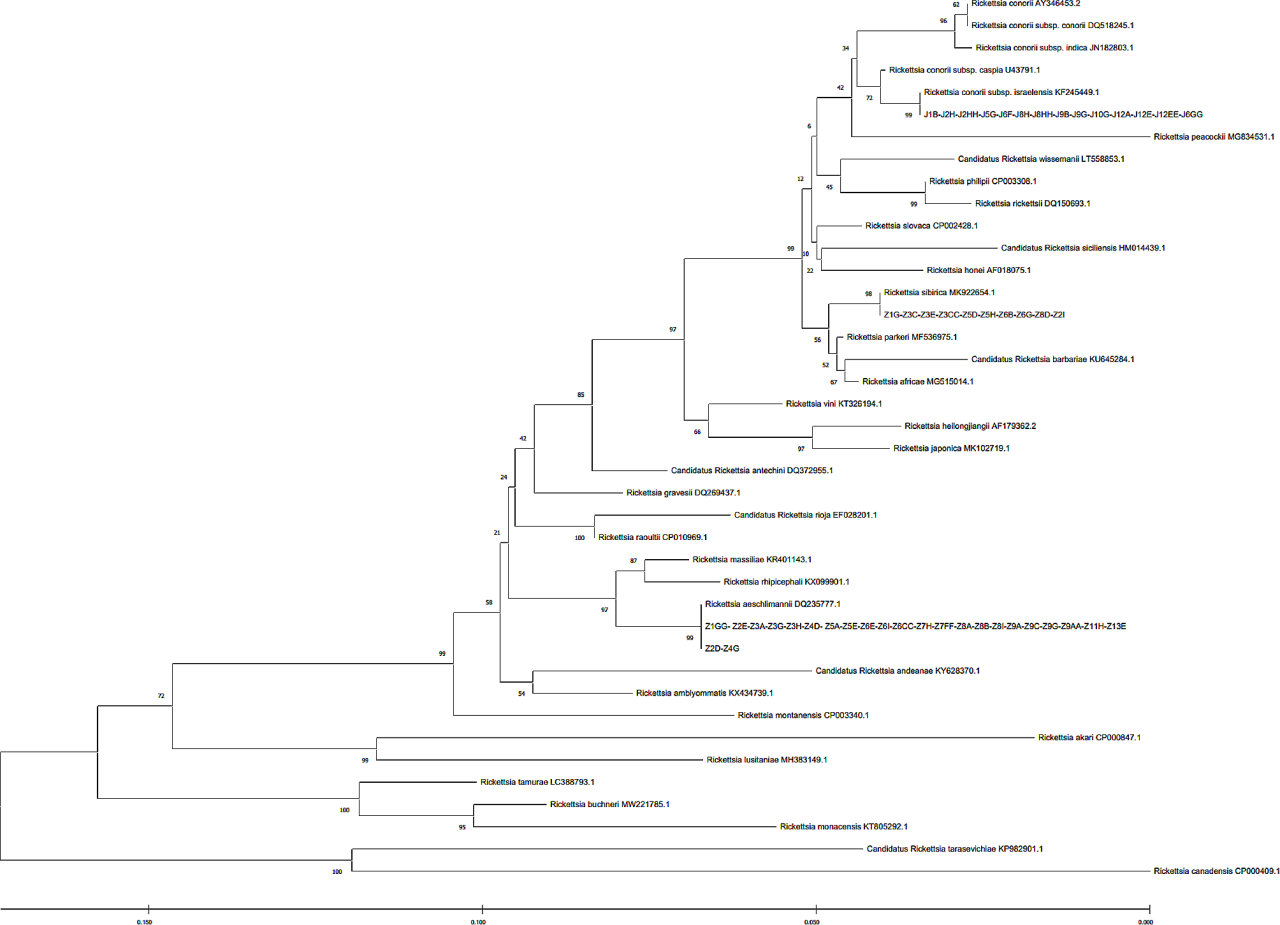
Phylogenetic tree diagram of *ompA* gene, extracted from bioinformatics analysis

The prevalence of *R. conorii israelensis* was 28.57% (28.57%; 95% CI 15.92–41.22) of 49 sequenced positive samples in Kerman Province (Jiroft County only). According to sequencing and BLAST analysis in GenBank, the *gltA* gene sequence in all positive samples for *R. conorii israelensis*, except for the J6GG sample, was identical (matched 100%) to each other, with 100% similarity with the sequence of human clinical cases reported for this bacteria from Kerman Province. The J6GG sample had only one nucleotide difference in sequence with the other samples of *R. conorii israelensis* obtained in this study (Fig. 2). In addition, the sequence obtained for the *ompA* gene for all positive samples of *R. conorii israelensis* was exactly similar to each other and had 100% similarity (matched 100%) with the sequence of human clinical cases reported for this bacterium from Kerman Province (Fig. 3).

The prevalence of *R. sibirica* was 20.41% (20.41%; 95% CI 9.12–31.70) of 49 sequenced positive samples in Kerman Province (Zarand County only). According to the sequencing and BLAST analysis in GenBank, the obtained sequences of the *gltA* gene of all samples related to this species (Z1G, Z3C, Z3E, Z3CC, Z5D, Z5H, Z6B, Z6G, and Z8D), except for the Z2I sample in this study, had the same sequence (matched 100%) (Fig. 2). In addition, according to sequencing and analysis of the *ompA* gene sequence, all *R. sibirica* samples in this study were similar to each other (matched 100%) (Fig. 3).

The prevalence of *R. aeschlimannii* was 48.98% (48.98%; 95%CI 34.99–62.97) of 49 sequenced positive samples in Kerman Province (Zarand County only). According to the sequencing and sequence analysis of the *gltA* gene, the sequences of all samples of *R. aeschlimannii* in this study were exactly similar (matched 100%) to each other (Fig. 2). Also, Z4G and Z2D samples had 100% identical sequences in *ompA* gene and sequences of these two samples had very little different from other identified *R. aeschlimannii* in this study (Fig. 3).

The prevalence of *R. helvetica* was 2.04% (2.04%; 95% CI 0–6) in the 49 sequenced positive samples from Kerman Province (Zarand County only). The sequence of a single positive sample of *R. helvetica* was the same as that recorded for the *gltA* gene in GenBank (100%match) (Fig. 2). In addition, due to the absence of the *ompA* gene in *R. helvetica*, the amplification result for single positive sample in our study was negative (Fig. 3).

The results showed a significant association between county variables and tick spp. (*p* < 0.001), *Rickettsia* genus infection in ticks (*p* < 0.001) and *Rickettsia* spp. infection (*p* < 0.001). In addition, there was a significant association between tick species. variable in association with host animals (*p* < 0.001), *Rickettsia* genus infection in ticks (*p* < 0.001), and *Rickettsia* spp. (*p* < 0.001) variables were observed. However, no significant association was observed between the host animal variable and *Rickettsia* genus infection in ticks (*P* = 0.076 > 0.05) or the host animal variable in association with *Rickettsia* spp. in ticks (*P* = 0.569 > 0.05).

The evolutionary history was inferred using the Neighbor-Joining method. The optimal tree with the sum of branch length = 1.15024671 is shown. The percentage of replicate trees in which the associated taxa clustered together in the bootstrap test (1000 replicates) are shown next to the branches. The tree is drawn to scale, with branch lengths in the same units as those of the evolutionary distances used to infer the phylogenetic tree. The evolutionary distances were computed using the Kimura 2-parameter method and are in the units of the number of base substitutions per site. The rate variation among sites was modelled with a gamma distribution (shape parameter = 1). This analysis involved 40 nucleotide sequences. All positions with less than 95% site coverage were eliminated, i.e., fewer than 5% alignment gaps, missing data, and ambiguous bases were allowed at any position (partial deletion option). There were a total of 462 positions in the final dataset. Evolutionary analyses were conducted in MEGA X.

The evolutionary history was inferred using the Neighbor-Joining method [1]. The optimal tree with the sum of branch length = 0.53847388 is shown. The percentage of replicate trees in which the associated taxa clustered together in the bootstrap test (1000 replicates) are shown next to the branches [2]. The tree is drawn to scale, with branch lengths in the same units as those of the evolutionary distances used to infer the phylogenetic tree. The evolutionary distances were computed using the Kimura 2-parameter method [3] and are in the units of the number of base substitutions per site. The rate variation among sites was modeled with a gamma distribution (shape parameter = 1). This analysis involved 45 nucleotide sequences. Codon positions included were 1st + 2nd + 3rd + Noncoding. All positions with less than 95% site coverage were eliminated, i.e., fewer than 5% alignment gaps, missing data, and ambiguous bases were allowed at any position (partial deletion option). There were a total of 747 positions in the final dataset. Evolutionary analyses were conducted in MEGA X [4].

## Discussion

*Rickettsia* occurs worldwide and rickettsiosis is recognized as an emerging infection in several parts of the world. Few studies have been conducted on the identification of tick fauna in Kerman Province. In our study, among the 2100 ticks collected, 1128 belonged to *Rhipicephalus linnaei* and 972 belonged to *Hyalomma deteritum*. In a study (2008–2009), was investigated the prevalence of hard ticks in cattle and sheep in southeastern Iran, *Rhipicephalus* and *Hyalomma* ticks have been identified as dominant ticks. A comparison of the results showed that *Rhipicephalus* and *Hyalomma* ticks were dominant in southeastern Iran. In addition, the difference in the species of these ticks may be due to, the large spread and vastness of rural areas in Kerman Province [14].

According to the results, the presence of the *Rickettsia* genus was observed in 24.9% (95%CI 20.28–29.52) of 350 samples. Sequencing and phylogenetic analyses revealed the presence of *R. aeschlimannii* (48.98%), *R. conorii israelensis* (28.57%), *R. sibirica* (20.41%), and *R. helvetica* (2.04%) in positive samples. In a similar study, Mostafavi et al. (2019–2020), reported a 20% prevalence of *Rickettsia* in ticks of stray dogs in Kerman city and the presence of *Rickettsia* spp. including *R. massiliae*, *R. rhipicephali*, and *R. sibirica* in *Rhipicephalus sanguineus sensu lato* ticks. Since both studies were conducted in Kerman Province, it can be concluded that the prevalence of *Rickettsia* in ticks was significant, therefore the prevalence of *Rickettsia* in our study was relatively higher, and the prevalence of *R. sibirica* in ticks in both studies was significant [15].

To date, few studies have been conducted on the prevalence and species of *Rickettsia* in domestic animals and dog ticks in Iran. In 2020, the prevalence of SFG *Rickettsia* in ticks collected from domestic animals and birds in nine provinces of Iran was 59%. The prevalence of *rickettsia* in this study was higher than that in our study, which could be due to the extent of the studied areas and the diversity of tick species [16]. In one study, a 50% prevalence of *Rickettsia* was observed in hard ticks collected from sheep in nine counties of the Khuzestan Province of Iran. According to sequencing and phylogenetic analyses, a significant presence of *R. aeschlimannii* (60%), *R. massiliae* (30%), and *Rickettsia conorii* (10%) was detected in infected ticks. The prevalence of *Rickettsia* in this study is higher than in our study, which could be due to extent of the studied areas and the diversity of tick species, compared to our study. However, the similarity between the two identified species, *R. aeschlimannii* and *R. conorii*, in both studies indicates the prevalence of these two species in southern Iran [5].

Based on a recent study in Iran’s northern provinces (Guilan, Mazandaran, and Golestan), 25.2% of collected ticks were positive for *Rickettsia*, and the 8 species of *Rickettsia* were identified including *R. massiliae, R. sibirica*, *R. rhipicephali, R. aeschlimannii*, *R.helvetica, R. asiatica*, *R. monacensis*, and *R. raoultii*. The similarity of the three species of *Rickettsia* (*R.sibirica, R. aeschlimannii and R. helvetica*) identified in this study with our study indicates the prevalence of these species in northern and southern Iran [17].

In other countries, the prevalence of *Rickettsia* in ticks in Pakistan 14% [18], in Italy 18.4% [19], in Ukraine 19.1% [20] and in Turkey 1.9% [21] has been reported. A comparison of the results of these studies with those of our study showed a significant prevalence of *Rickettsia* (24.9%). this could be due to differences in climatic conditions, the diversity of tick species, and an increase in the population of ticks in our study areas. However, in other studies, the prevalence of *Rickettsia* in ticks: in Italy at 52.25% [22], in Italy at 33% [23], Ghana at 45.6% [24], and France at 25.6% [25] has been reported. In these studies, a significant prevalence of *Rickettsia* compared to our study has been reported, which could be due to the favorable climatic conditions for the growth of ticks, diversity of tick species involved in the reproduction and transmission of *Rickettsia*, animal tick contamination, and increased tick populations.

In the present study, two species of *R. aeschlimannii* and *R. conorii israelensis* had the highest prevalence, both of which are members of SFG *Rickettsia*. *R. aeschlimannii* is a tick-borne *Rickettsia* that is known as a pathogenic species in Europe and Africa [26]. *R. aeschlimannii* is associated with cases (diseases) similar to Mediterranean spotted fever (MSF) in Africa and is distributed in Mediterranean areas [27]. *R. conorii* is responsible for MSF, and *Rhipicephalus sanguineus* tick is considered the main vector [28]. Similar to our findings, in Italy, *R. conorii israelensis* from *Rhipicephalus sanguineus* ticks (17.6%; 95%CI 4.67–44.20) and *R. aeschlimannii* from *Hyalomma marginatum marginatum* ticks (8.3%; 95%CI 0.44–40.25) has been reported [29]. Another study in Italy reported a 33% prevalence of *Rickettsia* in ticks. In this study, *R. aeschlimannii* was identified in *Hyalomma marginatum* and *Hyalomma lusitanicum* ticks, and *R. conorii* was identified in *Rhipicephalus sanguineus* ticks [23]. In Turkey, 41% prevalence of *Rickettsia* in human ticks, *R. aeschlimannii* in *Hyalomma marginatum, Hyalomma aegyptium* ticks (12%), *R. conorii conorii* in *Rhipicephalus bursa* ticks (4%), and *R. helvetica* in *Ixodes ricinus* ticks (2.3%) has been reported [30]. Considering the prevalence of *R. aeschlimannii* and *R. conorii* in these areas and our study, it is recommended that the health system pay attention to the dangers of their spread.

The other *Rickettsia* spp. identified in our study were *R. sibirica* and *R. helvetica*, which were relatively less common. These two species were identified only in ticks from Zarand County. *R. helvetica* is classified as a pathogenic species in SFG *Rickettsia* [26]. *R. helvetica* is also involved as a human pathogen with fever, with or without rash, and in patients with meningitis and carditis [27]. Siberian tick-borne typhus (STT) is caused by *R. sibirica*, which was previously reported to be the only tick-borne rickettsiosis agent in the Asian part of Russia [31]. Lymphangitis-associated rickettsioses (LAR), caused by *R. sibirica mongolotimonae*, have been recognized in various European countries (France, Spain, Portugal, and Greece) [27].

Similar to our findings, in the Asian part of Russia, *R. sibirica* (12.1%) was detected in *Dermacentor nuttalli* ticks and *R. helvetica* (1.9%) in *Ixodes persulcatus* ticks [32]. In another study in Spain, a 17.6% prevalence of *Rickettsia* in ticks was reported. Additionally, *R. sibirica* (1.12) and *R. helvetica* (1.12) have been identified in *Ixodes ricinus* ticks [33]. In Sweden, the prevalence of *Rickettsia* in ticks was reported to be 9.54–9.6%. In addition, *R. sibirica* and *R. helvetica* (with the highest amounts) were detected in *Ixodes ricinus* ticks [34].

Our study and several published studies in Iran indicate the existence of different species of *Rickettsia*. Therefore, it is possible to identify these species by conducting extensive and comprehensive studies. The results of the present study showed a significant association between county variables and the following variables: tick spp. (*p* < 0.001), *Rickettsia* infection in ticks (*p* < 0.001) and *Rickettsia* spp. (*p* < 0.001). In addition, a significant association between tick species and host animals (dogs and domestic animals) (*p* < 0.001), *Rickettsia* infection in ticks (*p* < 0.001), and *Rickettsia* spp. (*p* < 0.001) was observed, because all ticks collected from Jiroft County belonged to *Rh. linnaei*, whereas in Zarand County *Rh. linnaei* was collected only from dogs, and H. deteritum was collected only from domestic animals. The prevalence of *Rickettsia* in ticks from Zarand County (38.9%) was higher than that in the ticks from Jiroft County (10%). *Rickettsia* spp. isolated from ticks in Zarand County (*R. aeschlimannii*, *R. sibirica*, and *R. helvetica*) differed from those isolated from ticks in Jiroft County (*R. conorii israelensis*). There were differences in the parasitization of animals by specific genera and species of ticks. For example, only *Rh. linnaei* was collected from dogs. *Rickettsia* infection in *H. deteritum* ticks (37.66%) was higher than in *Rh. linnaei* ticks (13.80%). *Rickettsia* spp isolated from *Rh. linnaei* and *H. deteritum* ticks were different. For example, *R. conorii israelensis* has been isolated only from *Rh. linnaei* ticks.

There was no statistical association between the host animal variables and the following variables: *Rickettsia* genus infection and *Rickettsia* spp. indicating a lack of a role for the host animal (dogs and domestic animals) in the prevalence of *Rickettsia* and its species in Kerman Province.

Our limitations in this study were the impossibility of collecting samples from more counties of Kerman Province. In addition, because of the possibility of the prevalence of *Rickettsia* in a wide range of ticks of birds and animals (domestic and wild) in Kerman Province, we could not solve these limitations due to the lack of facilities and time. Therefore, future studies should investigate the prevalence of *Rickettsia* spp. in wider areas and more animal ectoparasites.

## Conclusion

According to the findings of this study, it is recommended that the health system be informed about *Rickettsia* species circulating in these areas. Therefore, to better understand the epidemiological situation of rickettsiosis in Iran, more studies should be conducted in the field of detection of *Rickettsia* species in animals and their external parasites (especially ticks and fleas), as well as a detailed investigation of suspected human cases in different regions of Iran.

## Data Availability

All data generated or analyzed during this study are included in this published article.
